# Quorum Sensing Inhibition by *Asparagopsis taxiformis*, a Marine Macro Alga: Separation of the Compound that Interrupts Bacterial Communication

**DOI:** 10.3390/md11010253

**Published:** 2013-01-23

**Authors:** Bhavanath Jha, Kumari Kavita, Jenny Westphal, Anton Hartmann, Philippe Schmitt-Kopplin

**Affiliations:** 1 Discipline of Marine Biotechnology and Ecology, CSIR—Central Salt and Marine Chemicals Research Institute, G. B. Marg, Bhavnagar 364002, Gujarat, India; E-Mail: kkavita@csmcri.org; 2 Research Unit Analytical Biogeochemistry, Helmholtz Zentrum München, Ingolstaedter Landstrasse 1, D-85764 Neuherberg, Germany; E-Mails: jenny.westphal@helmholtz-muenchen.de (J.W.); schmitt-kopplin@helmholtz-muenchen.de (P.S.-K.); 3 Research Unit Microbe-Plant Interactions, Helmholtz Zentrum München, Ingolstaedter Landstrasse 1, D-85764 Neuherberg, Germany; E-Mail: anton.hartmann@helmholtz-muenchen.de; 4 Analytical Food Chemistry, Technische Universität München, Alte Akademie 10, D-85354 Freising-Weihenstephan, Germany

**Keywords:** marine algae, quorum sensing inhibitor, acyl homoserine lactone (AHL), *Chromobacterium violaceum*, solid phase extraction

## Abstract

The majority of the marine algal species, though completing their life cycle in seawater, are rarely susceptible to fouling, making them an important source of quorum sensing (QS) inhibitory substances. The separation and characterization of QS inhibitors are crucial for any potential application. Thirty marine macroalgae were tested for QS inhibition activity by using *Chromobacterium violaceum* CV026 as the reporter strain, and among them, *Asparagopsis taxiformis* showed antibacterial, as well as antiquorum, sensing activities. Cinnamaldehyde (75 mM) and methanol were used as positive and negative controls, respectively. The antiquorum sensing activity of *A. taxiformis* was further confirmed using the sensor strain, *Serratia liquefaciens* MG44, having green fluorescent protein (gfp). Methanolic extract of the alga was fractionated by solid phase extraction (SPE), and each fraction was tested for QS inhibition. Two types of activities were observed—zone of clearance (antibacterial activity) and zone of inhibition with or without finger-like projections (QS inhibition). Out of five SPE cartridges, Bond Elut PH showed clear separation of these two fractions. The Ion Cyclotron Resonance Fourier Transformation Mass Spectrometer (ICR-FT/MS) analysis of the fractions further supported the bioassay results. The presence of strong QS inhibitory compound in *A. taxiformis* indicates its potential use in antifouling preparations.

## 1. Introduction

Biofilms are organized communities of microbes sheathed in extracellular polymeric substances (EPS). It confers antibiotic resistance to the bacteria. The biofilm formation is controlled by quorum sensing [1]. Quorum sensing is a population density-dependent gene regulation through extracellular signaling molecules produced by bacteria. Many pathogenic bacteria apply quorum sensing as regulatory mechanism for their pathogenicity and production of virulence factor [2]. Therefore, quorum sensing can be used as an ideal antipathogenic drug target instead of antimicrobials, which lead to emergence of drug resistance in bacteria [3]. It has been found that quorum sensing inhibitors increase the susceptibility of bacterial biofilms to antibiotics *in vitro* and *in vivo* [4]. This led the interest of the scientific community to concentrate on quorum sensing inhibitors.

Biofilm causes adverse effects in several important structures, including water-supplying pipes, air ducts, catheters and fermenters in industries. It also causes erosion, clogging and slippery coatings on the surface, as well as harmful contamination of bacteria [5,6]. Marine biofouling is one of the major causes of economic loss to maritime industries [7,8]. Traditional antifouling paints containing toxic metals (copper, lead, mercury, arsenic, *etc.*) were replaced by organotins, considered to be most effective antifouling agents known, but they are highly toxic [9]. Organotins are non-biodegradable and have long-term effects. The International Maritime Organization (IMO) prevented their application in ships from September, 2008 onwards [9]. There is an urgent need for the development of ecofriendly and nontoxic antifouling compounds. Antifouling activity from several marine algae has been reported by Hellio and her co-workers [10,11,12,13,14].

Marine algae complete their entire life cycle in sea water, thus providing an ideal surface for biofilm formation. However, the occurrence of fully grown biofilm on marine algae is a rare event [15,16]. Marine algae are endowed with effective defense mechanisms to avert biofilm formation, such as quorum sensing inhibition through metabolite production. The first quorum sensing inhibitory compound was isolated from a red macro alga, *Delisea pulchra* [17], and its role in AHL regulatory systems and quorum sensing inhibition were shown [18,19]. However, there is a need for screening more seaweed for identification of novel quorum sensing inhibitors that can act as antifouling compounds [20].

In the present study, thirty marine algal extracts were tested for their quorum sensing inhibitory potential by using reporter strain *Chromobacterium violaceum* CV026. The *C. violaceum* CV026 is a mutant strain incapable of producing AHL and violacein. It is a versatile and easy-to-use reporter that responds to exogenous AHLs and is widely used in quorum sensing inhibition assay [21,22,23,24]. The strain is also used for checking antibacterial activity [25]. The extract that showed growth inhibition, as well as quorum sensing inhibition, was further fractionated using five different solid phase extraction (SPE) cartridges, and two distinct activities, antibacterial and quorum sensing inhibition, could be separated. The fractions were further subjected to ICR*-*FT*/*MS analysis. This is the first report of the quorum sensing inhibition property of *Asparagopsis taxiformis *and the capability of different SPE cartridges to separate quorum sensing inhibitor and antibacterial compounds. 

## 2. Results and Discussion

### 2.1. Screening of Quorum Sensing (QS) Inhibition Activity from Seaweed Extracts

Thirty different marine macro algae (seaweed) belonging to three divisions were tested for quorum sensing inhibition using *Chromobacterium violaceum* CV026. Among them, *A. taxiformis* showed QS inhibition (Table 1). Violacein production is a quorum sensing regulated behavior in strain CV026. The white colored, opaque zone of inhibition with intact bacteria represents the QS inhibition. Antibacterial activity, represented by transparent zone/growth inhibition, was also observed. Cinnamaldehyde was used as a positive control, because at low concentrations, it does not inhibit the growth of the reporter strain and inhibits AHL-mediated QS [26,27]. The predicted mechanism of QS inhibition involves the interference of three carbon aliphatic side chains, with the binding of the smaller AHLs to their cognate receptors [26]. The biofilm formation is one of the important means of fouling in marine habitat. It is a quorum sensing-mediated process. QS controls bacterial biofilm differentiation and maturation, and its disruption may prevent microbial biofouling [20]. *A. taxiformis* belongs to red algae (Bonnemaisoniales, Bonnemaisoniaceae and Rhodophyta). It has been reported earlier that red algae show the highest antifouling activity among three groups of marine macro algae [28]. It is worth mentioning that *Delisea pulchra*, belonging to red algae, is known to synthesize halogenated furanones, which inhibit quorum sensing through accelerated LuxR turnover [17,18,19].

**Table 1:** List of algal samples (from all the three divisions: Chlorophyta, phaeophyta and rhodophyta) screened for quorum sensing (QS) inhibition activity against *Chromobacterium violaceum* CV026. The bioassay was performed in triplicates. Methanol was used as negative control, and cinnamaldehyde was used as positive control.

Serial No.	Name of Algae	Division	Quorum sensing inhibition
1.	*Padina gymnospora*	Phaeophyta	Negative
2.	*Sargassum wightii*	Phaeophyta	Negative
3.	*Pocockiella variegate*	Phaeophyta	Negative
4.	*Turbinaria ornate*	Phaeophyta	Negative
5.	*Stoechospermum marginatum*	Phaeophyta	Negative
6.	*Cystoseria trinodis*	Phaeophyta	Negative
7.	*Sargassum myriocystum*	Phaeophyta	Negative
8.	*Sargassum ploiophyllum*	Phaeophyta	Negative
9.	*Asparagopsis taxiformis*	Rhodophyta	Positive
10.	*Chondrococcus harnemanii*	Rhodophyta	Negative
11.	*Gracilaria edulis*	Rhodophyta	Negative
12.	*Hypnea pannosa*	Rhodophyta	Negative
13.	*Jania adhaerens*	Rhodophyta	Negative
14.	*Hypnea valentiae*	Rhodophyta	Negative
15.	*Pterocladia heteroplatos*	Rhodophyta	Negative
16.	*Galaxaura obtuse *	Rhodophyta	Negative
17.	*Halicrysis tchivye*	Rhodophyta	Negative
18.	*Acanthophora spicifera*	Rhodophyta	Negative
19.	*Champia parvula*	Rhodophyta	Negative
20.	*Hypnea flagelliformis*	Rhodophyta	Negative
21.	*Chondracanthus acicularis*	Rhodophyta	Negative
22.	*Porphyra kanyakumariensis*	Rhodophyta	Negative
23.	*Polysiphonia tuticoriensis*	Rhodophyta	Negative
24.	*Laurencia papillosa*	Rhodophyta	Negative
25.	*Sarcodia ceylanica*	Rhodophyta	Negative
26.	*Chaetomorpha antennina*	Chlorophyta	Negative
27.	*Caulerpa veravalensis*	Chlorophyta	Negative
28.	*Cladophora indica*	Chlorophyta	Negative
29.	*Enteromorpha spp.*	Chlorophyta	Negative
30.	*Ulva fasciata*	Chlorophyta	Negative

**Figure 1:**
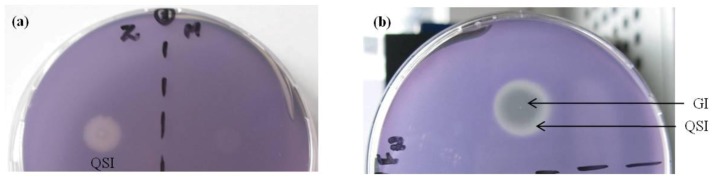
(**a**) Z: Cinnamaldehyde as positive control and M: Methanol as negative control; (**b**) Antibacterial and antiquorum sensing activity by *A. taxiformis *extract; the central part shows growth inhibition (GI), indicating antibacterial activity, and the peripheral part shows Quorum sensing inhibition (QSI).

The cinnamaldehyde (positive control) showed only QSI, turbid growth and inhibition of violacein (Figure 1a). Standardization of cinnamaldehyde concentration showed maximum QS inhibition at 75 mM (data not shown). Methanol was used as negative control and did not have any significant effect on violacein production (Figure 1a). Antibacterial activity is revealed through a zone of clearance at the center, while QSI is seen at the periphery (Figure 1b). Some of the gorgonian corals from the Caribbean reef showed antibacterial and QS inhibitory effects [24]. Lyngbyoic acid obtained from a marine cyanobacterium is known to inhibit quorum sensing [29]. Since, *N*-acyl-homoserine lactone is considered as a signaling molecule of widely present Gram-negative bacteria in marine environment, we have targeted the LuxI/LuxR-type QS system of Gram-negative bacteria as a reporter system for the study of QS inhibitors from some common seaweeds. Though the antimicrobial potential of *Asparagopsis *had been reported earlier [30,31,32,33,34,35], we are reporting the quorum sensing inhibition for the first time.

### 2.2. Evaluation of *A. taxiformis* Extract Fractions Obtained from SPE Cartridges

**Figure 2:**
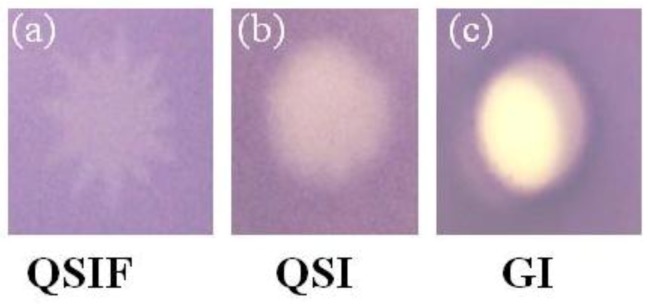
Quorum sensing inhibition activity of *A. taxiformis *extract fractions against: (**a**) quorum sensing Inhibition with finger-like projection (QSIF); (**b**) Quorum sensing Inhibition (QSI); (**c**) Growth inhibition (GI) showing fraction with antibacterial activity.

Five different SPE cartridges were used to fractionate the extract, and 50 different fractions were collected. C_2_ and C_18_ cartridges showed clear separation of two activities, one showing QSI and another antimicrobial activity (Table 2). In contrast, the fractions obtained using CN-E cartridge showed only antimicrobial activity, and those from modified styrene-divinylbenzene polymer (PPL) showed only QSI (with finger-like projections). The PH cartridge was most efficient in separating the fractions into QSI and antimicrobial activity (Table 2). The properties of the sorbents present in different cartridges help in predicting the structure (aliphatic/aromatic) and nature (polar/non polar) of the possible active compound (Table 3). Using PH cartridge, it was also possible to differentiate the fraction showing QSI into distinct finger-like projections (Figure 2a). The results clearly show that with antimicrobial activity, QSI and QSI with finger-like projections could be assigned to different fractions of the extract (Figure 2a–c). We presume that QSI and QSI with finger-like projections may be due to two different active compounds or the derivative of the same with a different diffusion. We could separate these two effects using different cartridges (Table 2). C2 cartridge gave only QSI, while in PPL, only QSI with finger-like projection was seen. This needs further validation by identifying the compounds. The variations obtained with different cartridges may be linked to the compounds with different polarities and their interactions with the sorbent (as detailed in the Experimental section). The Bond Elut PH exhibits slightly different selectivity from other non polar sorbents. This added selectivity results from the electron density of the aromatic ring present in it. Retention of planar conjugated organic molecules is enhanced as compared to aliphatic bonded phases.

**Table 2:** Summary of results of quorum sensing inhibition bioassay of fractions eluted using five different cartridges (Bond Elut C_2_, C_18_, CN-E, PH and PPL). The results of bioassay were expressed as: (+) denotes slight (faint) effect, + denotes minimal effect, ++ denotes medium effect, +++ denotes highest effect and − denotes absence of effect. “Fing” denotes finger like projection. The bioassays were performed in triplicates. SPE: solid phase extraction.

Bioassay results of fractions of *Asparagopsis taxiformis *extract											
Bond Elut SPE cartridges	Effects on plate based bioassay	Methanol (v/v)									
		10%	20%	30%	40%	50%	60%	70%	80%	90%	100%
C2	QS inhibition	(+)	−	−	(+)	−	−	(+)	−	−	+
	Zone of clearance	−	−	−	−	++	+++	−	−	−	−
C18	QS inhibition	+	−	−	−	−	+	−	−	+fing	(+)fing
	Zone of clearance	−	−	−	−	−	−	++	+++	−	−
CN-E	QS inhibition	−	−	−	−	−	−	−	−	−	−
	Zone of clearance	−	−	++	++	++	−	−	−	−	−
PH	QS inhibition	−	−	−	−	++	−	−	++	(+)fing	+fing
	Zone of clearance	−	−	−	−	−	++	++	−	−	−
PPL	QS inhibition	+fing	−	−	−	−	−	−	(+)fing	+fing	++fing
	Zone of clearance	−	−	−	−	−	−	−	−	−	−

**Table 3:** Characterization of used SPE cartridges for fractionation of extract of *A. taxiformis*. All cartridges were equipped with the following dimensions: 100 mg, 1 mL, 40 μm particle size.

Bond Elut SPE-Cartridges	Type of Material	Properties	Primary Retention Mechanism	Typical Sample Types
C_2_	Silica based, ethyl bonded, endcapped	Alternative sorbent, if analytes are retained too strongly on C_8_ or C_18_ phases	Weakly nonpolar	Plasma, urine, aqueous samples
C_18_	Silica based, trifunctional octadecyl bonded, endcapped	Extreme retentive nature for nonpolar compounds, applicable for desalting aqueous matrices	Strongly nonpolar	Water, aqueous biological fluids
CN-E	Silica based, cyanopropyl bonded, endcapped	Ideal sorbent for extracting extremely nonpolar compounds	Moderately nonpolar (aqueous matrix) or polar (nonpolar organic matrix)	Aqueous samples (nonpolar), organic samples (polar)
PH	Silica based, phenyl bonded, endcapped	Different selectivity to alkyl and aliphatic functionalized phases due to electron density of the aromatic ring	Moderately nonpolar	Water, biological fluids
PPL	Styrene-divinylbenzene (SDVB) polymer with a proprietary derivitized nonpolar surface	Extreme hydrophobicity and surface area, achieves high recovery levels and fast extraction speeds	Highly polar	Waste water (phenols)

**Figure 3:**
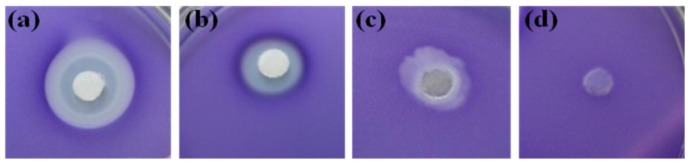
Disc diffusion assay for testing the antibacterial activity of the extract and fractions of *A. taxiformis *against strain CV026: (**a**) extract; (**b**) fraction showing GI; (**c**) fraction showing QS inhibition; and (**d**) control (methanol).

Further, the toxic effect of the extract and fractions on the reporter, *C*. *violaceum* CV026, was investigated using the disc diffusion method. The results are depicted in Figure 3. The extract gave both GI, a clear zone around the disc and QS inhibition at the periphery (Figure 3a). The fraction of the extract, which showed GI in QSI bioassay (Figure 2c), showed a clear zone, confirming the GI in the disc assay (Figure 3b). As expected, another fraction gave a white opaque zone around the disc (Figure 3c) without showing any GI. The result confirms the findings of QSI assay and also supports the notion that the QSI is based on the interference of bacterial signaling, rather than antibacterial activity.

### 2.3. Evaluation of Bioassay Using *Serratia liquefaciens* MG44

**Figure 4:**
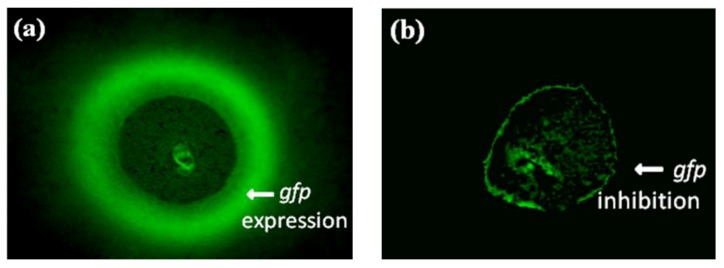
Inhibition of Green fluorescent protein (gfp) expression by algal extract (*Asparagopsis taxiformis*) (**a**) *Serratia liquefaciens* + *N*-acyl homoserine lactones (AHL); (**b**) *Serratia liquefaciens* + AHL + Extract (*Asparagopsis taxiformis*). The bioassay was performed in triplicate.

The QSI activity of the individual fraction of the extract was further confirmed using an isogenic AHL-negative strain (MG44) of *Serratia liquefaciens*. In the biosensor, *S. liquefaciens*, the sole AHL synthase gene *swrI* is mutated by gene replacement, and it gives a green fluorescent protein in presence of external AHL [36]. The absence of green color fluorescence indicates the quorum sensing inhibition. The positive fraction of the extract inhibited the AHL-induced green fluorescence (Figure 4). This confirmed the QSI of the extract as observed in the *C. violaceum* CV026 bioassay. 

### 2.4. Interpretation of ICR-FT/MS Analysis

**Figure 5:**
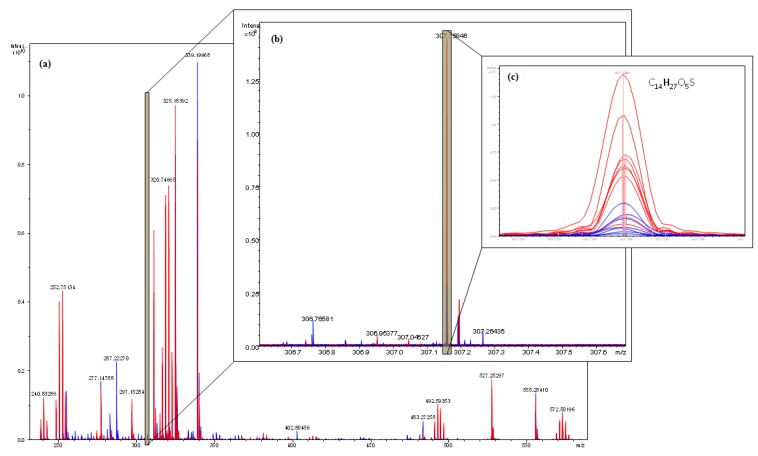
Fourier transform ion cyclotron resonance mass spectrometry (ICR-FT/MS) spectra of SPE extracts overlaid (fractions showing antibacterial activity colored red, QS inhibition in blue). (**a**) Represents full spectra; (**b**) Zoomed into one nominal mass; and (**c**) zoomed into one single signal.

ICR-FT/MS has a molecular mass resolution enabling to overlay all spectra of fractions showing antibacterial activity from the various SPE systems in one color (red) together with the adjacent non-active (QS inhibition) fractions (in blue) to constrain the positive hits corresponding to the active compounds (Figure 5a–c). Overlaid ICR-FT/MS spectra showed considerable analogies between *A. taxiformis* fractions, which were tested positive for antibacterial activity, and those who were for QS inhibition, except for a few signals. For some detected masses, the intensities were significantly increased for many active fractions. The ultrahigh resolution of the high field ICR-FT/MS system enabled a direct assignment of elementary composition to the exact masses of interest with a 200 ppb precision. Figure 5c showed a peak corresponding to a molecular mass of 307.58. The likely chemical formula of the compound is predicted to be C_14_H_27_O_5_S, and the expected active compound could be 2-dodecanoyloxyethanesulfonate. The calculated molecular mass of the compound is 307.4, which is close to the observed mass. To our best of knowledge, the molecular weight does not resemble any of the previously reported inhibitors. However, sulfur-containing AHL-analogues, *N*-(propylsulfanylacetyl)-L-homoserine lactone and *N*-(pentylsulfanylacetyl)-L-homoserine lactone are known to inhibit QS [37].

## 3. Experimental Section

### 3.1. Collection and Extract Preparation of Algal Samples

Thirty different seaweed samples were collected from intertidal zone of Bay of Bengal and Arabian Sea at six different locations from Tamil Nadu, India (Thonithurai, Nochiurani, Kovalam, Erwadi, Rameshwaram and Sadamunian Valasai with N 09°17′16.9″; E 79°11′11.4″, N 09°16′16.0″; E 79°01′02.0″, N 08°05′05.3″; E 77°33′33.3″, N 09°12′12.4″; E 78°43′43.5″, N 09°09′09.6″; E 78°39′39.5″, N 09°11′11.4″; E 78°43′43.1″, respectively). The herbaria have been deposited in the Taxonomic Reference Center for Seaweeds at CSIR-CSMCRI, Bhavnagar, India. The fresh samples were washed twice with seawater. The samples were sterilized using ethanol (50%) and sodium hypochloride (1%) [38]. Thereafter, washed samples were blot dried and methanol extract was prepared from each freeze-dried sample (1 g/10 mL). The powdered algal samples were stirred for 24 h. The seaweed extracts were filtered twice (Whatman filter paper no. 1) and then concentrated (1 g/10 mL) under vacuum using a rotary evaporator (BÜCHI, Switzerland) at low temperature (35 °C) to avert evaporation of volatile compounds [33]. The concentrated extracts were stored at −20 °C for further analysis.

### 3.2. Bioassay for Quorum Sensing Inhibition (QSI)

Production of violet color pigment is a quorum sensing regulated behavior in *C. violaceum *strain CV026. The extracts were screened for QSI using *C. violaceum *CV026 as the reporter strain, as per Milton *et al*., with modification [23,39]. Five hundred microliters of overnight grown culture of *C. violaceum* CV026 was inoculated in 20 mL of nutrient broth medium and incubated at 30 °C, shaking at 170 rpm for 2–3 h until the optical density reached up to 0.7 at 600 nm. Nutrient broth soft agar (0.8% agar, 150 mL) was maintained at 45 °C, and 10 mL of culture (OD_600nm_ = 0.7), 10 μL from 1 mg/mL stock solution of hexanoyl homoserine lactone (Sigma-Aldrich, St. Louis, MO, USA) were added before plating. Five microliter extract was spotted on top of the agar. After overnight incubation of the plates at 30 °C, the surroundings of the spots were examined for inhibition of reporter violacein. Cinnamaldehyde (Sigma-Aldrich, St. Louis, MO, USA) was used as positive control, and the optimum concentration showing maximum QS inhibition was determined. Equal volume (5 μL) of methanol was used as negative control. Bioassay was performed in triplicate.

### 3.3. Fractionation of Extract

The methanol extract of *A. taxiformis *was dried and redissolved in purified, acidified water (pH-2.0). This exchange was needed to reverse the polarity of the samples and transfer them to solid phase extraction (SPE). The extracts were fractionated using five different SPE cartridges in changing polarity (Table 3), namely Bond Elut C_2_, C_18_, CN-E, PPL and PH (Agilent, Santa Clara, CA, USA) [40]. These cartridges have a volume of 1 mL, including 100 mg sorbent material of 40 μm particle size. SPE materials were chosen to be orthogonal in their sorption behavior to obtain a wide spectrum of analytes and classify them according to their hydrophobicity. 

Each cartridge was conditioned with 1 mL methanol, 5 mL purified water and 1 mL purified, acidified water (pH of 2.0) before loading with 200 μL of sample. The cartridges were than eluted consequently 10 times with 200 μL eluent of different methanol concentrations (from 10% to 100% v/v methanol in water). In total, 50 fractions (10 per cartridge) were collected.

### 3.4. Agar-Based Bioassay of Fractions

Each fraction was re-screened for QS inhibition using *C. violaceum* CV026, as given in Section 3.2 above. Results were analyzed by observing the surrounding of the spot. Veselova *et al.* [41] found that production of the purple pigment violacein is controlled by the quorum sensing molecule *N*-hexanoyl-L-homoserine lactone (HHL). *C. violaceum* 026 (CV026) is a mutant strain that is incapable of producing HHL and violacein. However, it possesses the capacity of violacein production in the presence of external AHLs, which can be evaluated by the color change of colony (white to purple) [41].

### 3.5. *Serratia liquefaciens* MG44 Bioassay

Quorum sensing inhibition activity of *A. taxiformis *was also confirmed using green fluorescence protein (GFP)-tagged *Serratia liquefaciens* MG44 as the reporter strain [36]. Overnight grown culture (150 μL) of *Serratia liquefaciens* MG44 in 5 mL Luria-Bertani (LB) medium, containing ampicillin (100 μg/mL), kanamycin (50 μg/mL) and tetracycline (20 μg/mL), was mixed with 50 μL of *N*-hexanoyl-L-homoserine lactone (0.5 mM) and spread on LB plate and kept for 30 min. Methanolic algal extract of *A. taxiformis *(5 μL) was spotted and kept for overnight incubation at 30 °C. The antiquorum sensing activity of the extract was observed under UV light.

### 3.6. ICR-FT/MS Analysis

Selected SPE fractions were analyzed by a 12 Tesla Ion Cyclotron Resonance Fourier Transformation Mass Spectrometer (ICR-FT/MS; Bruker, Bremen, Germany) coupled to an Apollo II Electrospray Ionization source (ESI; Bruker, Bremen, Germany) in negative mode. The samples were injected at a flow rate of 120 μL/h with a nebulizer gas pressure of 17.4 psi and a dry gas flow rate of 4 L/min (200 °C). All spectra were acquired with a collision energy of 1.5 V and a time domain size of 4 MWord within a mass range of 100–1000 *m*/*z*. For each spectrum, 100 scans were accumulated. All fractions showing antibacterial activity and each fraction, extracted with a methanol concentration less and above, were diluted 1 to 50 with methanol and transferred to a Gilson Auto sampler 223 (Gilson Incorporated, Middleton, Wisconsin, WI, USA). In total, spectra of 17 extracts were acquired.

## 4. Conclusions

Thirty different marine macro algae were screened for quorum sensing inhibition (QSI) activities using *Chromobacterium violaceum* CV026 as the biological reporter. Among them, *Asparagopsis taxiformis*, showed positive activities, including both QSI and growth inhibition (GI), which was confirmed to be assigned to different fractions. The effect of the QSI of the extract was also confirmed in *Serratia liquefaciens* MG44 with a gfp construct. Marine algae may produce QS inhibitory compounds as a safeguard against biofilm formation. The Bond Elut PH cartridge was most efficient to separate the extract into QSI and GI fractions. The ICR-FT/MS data indicated that the expected active compound could be 2-dodecanoyloxyethanesulfonate (C_14_H_27_O_5_S) with a calculated molecular mass of 307.4. Further experiments are required for the characterization and structural elucidation of the positive fractions.
